# Polysomnographic parameters in long-COVID chronic insomnia patients

**DOI:** 10.1080/19585969.2023.2222714

**Published:** 2023-06-30

**Authors:** Alexandre Rouen, Jonathan Taïeb, Gabriela Caetano, Victor Pitron, Maxime Elbaz, Dominique Salmon, Damien Leger

**Affiliations:** a Université Paris Cité, VIFASOM, ERC 7330, Paris, France; b AP-HP, Hôtel-Dieu, Centre du Sommeil et de la Vigilance, Paris, France; c Unité INSERM U933, Sorbonne Université, Paris, France; d Département de Génétique Médicale, Hôpital Armand-Trousseau, AP-HP, unité INSERM U933, Paris, France; e Université de Paris Cité, Paris, France; f Service Maladies Infectieuses et Tropicales, Hôpital Cochin Hôtel Dieu, AP-HP, Paris, France

**Keywords:** Long-COVID, sleep, insomnia, polysomnography

## Abstract

**Introduction:**

While COVID-19 is predominantly considered to be an acute self-remitting disease, it has been pointed out that a variety of symptoms can linger for several months, a phenomenon identified as long-COVID. Insomnia is particularly prevalent in long-COVID. In the present study, we aimed at confirming and characterising insomnia in long-COVID patients through polysomnography and to identify whether its parameters differ from patients with chronic insomnia and no long-COVID history.

**Materials and methods:**

We conducted a case-control study, including 17 long-COVID patients with insomnia symptoms (cases), and 34 2:1 matched controls with a diagnostic of chronic insomnia and no history of long-COVID. All underwent a one-night polysomnography (PSG).

**Results:**

First, we observed that long-COVID patients with insomnia complaints have altered PSG parameters, in favour of the diagnosis of chronic insomnia. Second, we show that insomnia related to long-COVID PSG parameters was not significantly different from regular chronic insomnia PSG parameters.

**Discussion:**

Our results indicate that even though it is one of the most prevalent symptoms of long-COVID, its related insomnia resembles typical chronic insomnia, based on PSG studies. Even though additional studies are warranted, our results suggest that the pathophysiology and therapeutic options should be similar to those recommended for chronic insomnia.

## Introduction

A novel coronavirus (SARS-CoV-2) was initially reported in China in December 2019, and its associated disease (COVID-19) was declared a Public Health Emergency of International Concern on 30 January 2020 by the World Health Organisation (WHO). As of 16 December 2022, 656,566,334 cases have been reported globally, with 6,668,035 deaths (COVID Live-Coronavirus Statistics-Worldometer n.d.).

In addition to the acute phase, numerous clinical manifestations have been reported in the weeks following COVID-19 infection, a phenomenon some authors called long-COVID, or post-acute sequelae of COVID-19 (PASC) (Barh et al. 2021). The World Health Organisation defines long-Covid as the continuation or development of new symptoms 3 months after the initial SARS-CoV-2 infection, with these symptoms lasting for at least 2 months with no other explanation (Soriano et al. 2022). The clinical symptoms observed in long-COVID are varied and affect different systems, including the nervous system (anosmia, headaches, ataxia, confusion, etc.), the skeletomuscular system (psoriasis, polyarthralgia), the gastrointestinal system (abdominal pain, nausea, diarrhoea, constipation, acid reflux, loss of appetite), the cardiovascular and pulmonary systems (myocardial hypertrophy, coronary artery disease, pulmonary thromboembolism, respiratory failure, pulmonary fibrosis), as well as mental health disorders (depression, anxiety, or insomnia). Symptoms most commonly reported are shortness of breath, fatigue, and cognitive dysfunction/brain fog (Al-Aly et al. 2021; Soriano et al. 2022).

Recently, the international COVID Sleep Study-II (ICOSS-II) aimed at evaluating sleep and wake disturbances in long-COVID (Merikanto et al. 2022). Merikanto et al. found that insomnia symptoms, fatigue, and excessive daytime sleepiness were among the most common complaints in long-COVID. Besides, there was a correlation between the prevalence of those symptoms and acute COVID-19 severity (Sudre et al. 2021).

Other studies on sleep disturbances in long-COVID were similarly based on subjective form-based data (Premraj et al. 2022; Young 2022). However, in long-COVID, there has been shown to be no correlation between subjective symptoms and functional limitations, hence the need for objective analyses (Ladlow et al. 2022). A recent study showed that chronic insomnia was the most common sleep-related complaint in long-COVID (Moura et al. 2022).

The study presented here is the first to our knowledge to use objective parameters from polysomnography to evaluate patients with long-COVID and sleep-related complaints. Additionally, we compared them to those of insomniac patients without any history of long-COVID, aiming to identify long-COVID-specific polysomnographic signs.

## Materials and methods

### Ethics

The survey has been approved by the Ethic committee (*CPP Ile de France 2*) under the reference 2018-05-06-RIPH 2° and the data were protected and anonymized according to the recommendations of the CNIL (*Comission Nationale Informatique et Liberté*).

### Protocol

A monocentric case-control survey with a double purpose:

1. Assessing objective sleep parameters in long-COVID patients with insomnia complaint (cases) by conducting polysomnographic studies,
2. Analysing whether polysomnographic parameters in insomniac long-COVID patients were significantly different from those in non-long-COVID patients with a diagnostic of chronic insomnia (controls) from the same sleep centre (Hôtel-Dieu, APHP, Sleep and Vigilance Sleep Centre, Paris, France).

### Subjects

Cases were 17 long-COVID patients with a complaint of chronic insomnia referred from the Infectious Diseases department to the Sleep department (Hôtel-Dieu, Paris, France), 6–18 months after an acute COVID infection. The diagnosis of chronic insomnia was also confirmed by a physician sleep specialist of the centre, according to the definitions of ICSD-3 and DSM-5 classifications of insomnia (APA 2013; International Classification of Sleep Disorders–Third Edition (ICSD-3) (Online)-American Academy of Sleep Medicine n.d.). Patients did not present with signs of other sleep disorders, such as sleep apnoea, periodic leg movements, or hypersomnia. None was night or shift worker, and none was on drugs affecting the central nervous system.

We documented the signs and symptoms presented by long-COVID patients in acute and chronic phase in Supplementary Table S1 and biological markers in Supplementary Table S2.

Controls (*n* = 34) were selected at the same period from the sleep department’s data file of insomniac patients who underwent a PSG around the same time period. Two controls were included for each case (2:1) and matched on age, sex, and body mass index. In our centre, we systematically record PSG in subjects with chronic insomnia according to the ICSD-DSM-5 criteria who consulted one of the sleep physicians of the centre.

### Polysomnography

PSG was performed according to the AASM guidelines and included: (i) at least three and usually six electroencephalographic (EEG) derivations at frontal (F3/F4), central (C3/C4), and occipital (O1/O2) sites and referenced to the contralateral mastoid, (ii) two electrooculography (EOG) derivations, and (iii) three electromyographic (EMG) derivations placed on the chin (*n* = 1) and legs (*n* = 2) (Sateia 2014). Respiratory parameters (respiratory flow, thoracic and abdominal bands, oxygen saturation), as well as body movements (position sensor and the two leg EMG derivations placed on the left and right tibial muscles), were also recorded to screen for obstructive sleep apnoea and periodic limb movement syndromes.

Classical scoring was performed first by a sleep physician and then by a sleep technician with more than 5 years of scoring experience, in accordance with the American Academy of Sleep Medicine (AASM) recommendations. The classification includes one wake stage (W) and four sleep stages: stage 1 (N1), stage 2 (N2), stage 3 (N3), and REM sleep (REM). Scoring is based on the visual analysis of 30-s periods of EEG, EMG, and EOG. Arousals are analysed as 3–15 s of acceleration of the EEG signals. Respiratory events included apnoea (90% respiratory flow decrease for more than 10 s) and hypopnoea (30–90% respiratory flow decrease associated with an arousal or oxygen desaturation of more than 3% and for more than 10 s). The central, obstructive, or mixed nature of the respiratory events was determined from the thoracic and abdominal bands signals. Periodic movement of the legs was scored based on four leg movements over a period of 1 min 30 s.

The following polysomnographic parameters of interest were considered: total sleep time (TST), sleep onset latency (SOL), wake after sleep onset (WASO), sleep efficiency (the ratio between total sleep time and total sleep period, expressed in percentage), rapid-eye-movement (REM) latency, duration of REM (expressed as a percentage of total sleep time), duration of N3 (expressed as a percentage of total sleep time), Apnoea Hypopnoea Index (AHI), Oxygen desaturation index (ODI).

The cases’ polysomnographic parameters are presented against normal values established for the adult population in a recent comprehensive meta-analysis (Boulos et al. 2019). These parameters are presented in a descriptive manner, without formal statistical analysis.

Visual inspection of the data with a Q–Q plot revealed obvious deviation from normality for all polysomnography parameters, except total sleep time and duration of REM. Non-parametric Wilcoxon signed-rank test and parametric *t*-test were used, accordingly, to compare polysomnography parameters between cases and controls.

### Statistics

All statistical analyses and data visualisation were performed with R (version 1.2.5019, R Development Core Team) (R Development Core Team 2012).

Unless otherwise specified, values are reported as mean and range (minimum–maximum), and *p*-values <0.05 were considered significant.

## Results

On first glimpse, the 17 long-COVID patients' PSG exhibited parameters indicative of insomnia (see Table 1): a short total sleep time >6 h (352 min), a normal sleep-onset latency (SOL <30 min), an important wake after sleep onset (WASO) of 83,2 min indication sleep maintenance insomnia (>30 min), a low sleep efficiency (SE), a high REM latency (>90 min) (with a normal REM duration), and a normal duration and proportion of slow wave sleep (stage N3). AIH values ranged between 0.8 and 38.9/h, with one only subject with results compatible with OSA.

**Table 1.** Polysomnography results for long-COVID patients with a complaint of insomnia (cases, *n* = 17) and patients with a diagnostic of chronic insomnia without a history of long-COVID (results with p<0.05 are considered statistically significant).

	Insomniac long-COVID cases	Insomniac controls	*p*-Value
	Mean (min–max)	Mean (IC 95%)	
Total sleep time (min)	352.5 (179.0–518.0)	334.5 (132.0–525.0)	0.46
Sleep latency (min)	26.7 (2.0–74.6)	32.7 (2.5–101.3)	0.52
Wake after sleep onset (min)	83.2 (25.0–198.5)	98.2 (9.4–305.5)	0.73
Sleep efficiency (%)	76.5 (48.5–93.2)	72.7 (30.2–96.3)	0.55
REM latency (min)	129.4 (58.0–238.0)	149.1 (0.0–416.5)	0.83
Duration of REM (percentage of total sleep time)	18.5 (3.3–38.2)	15.6 (0.0–32.1)	0.25
Duration of N3 (percentage of total sleep time)	21.1 (12.9–48.8)	25.8 (9.0–51.5)	0.05
Apnoea hypopnoea index (AHI)	8.9 (0.8–38.9) *	4.3 (0.0–11.6)	0.01
Oxygen desaturation index (ODI)	5.9 (0.4–38.5)	4.4 (0.0–31.6)	0.65

* = statistically significant.

Compared to the control group of chronic insomniacs which was matched with the long-covid patients, we found no statistically significant difference between the groups for any of the PSG parameters (TST, SOL, WASO, REM latency, REM percentage), except for the percentage of N3 which was higher in controls and the average AHI which was higher in subjects (Table 1).

**Figure 1.**
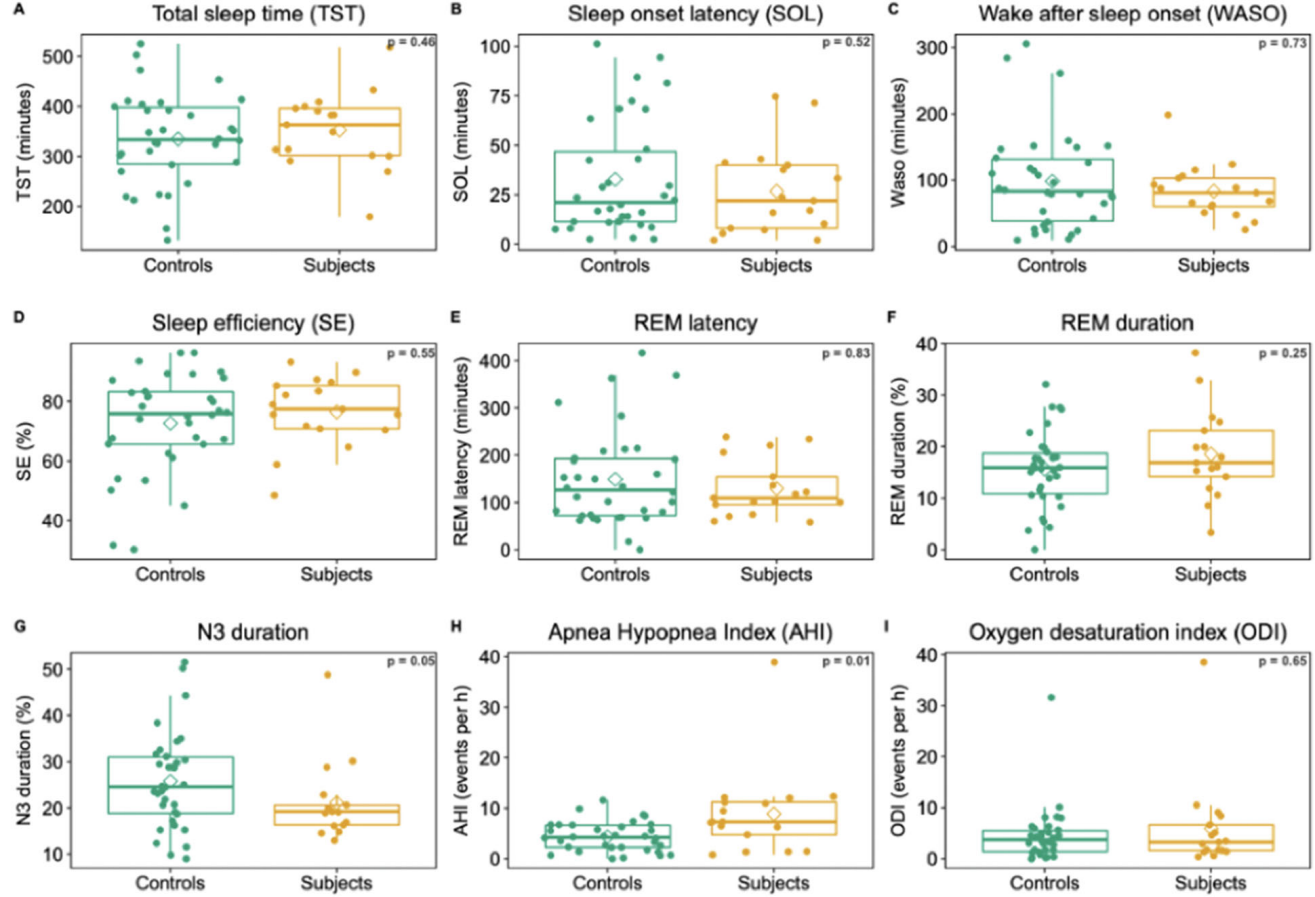
Polysomnographic parameters (box and whisker), for subjects and controls. The only statistically significant result (*p* = 0.01) is that of AHI, with higher values in subjects compared to controls.

Figure 1 provides details with box and whisker polysomnographic results for long-COVID patients with a complaint of insomnia (cases, *n* = 17) and controls with a diagnostic of chronic insomnia without a history of long-Covid (cases, *n* = 34).

## Discussion

Almost three years after the start of the COVID-19 pandemic, the persistence of symptoms in some patients long after contagion has raised interest. While, in most cases, COVID-19 is an acute disease, it quickly appeared that it could also be chronic, a phenomenon called long-COVID (or post-acute sequalae of COVID-19) (Callard and Perego 2021). Interestingly, similar post-viral syndromes were observed with other coronaviruses, such as severe acute respiratory syndrome (SARS) and Middle East respiratory syndrome (MERS) (Lam et al. 2009; Das et al. 2017).

The pathophysiology of long-COVID remains elusive. One possible mechanism is long-term tissue damage. Indeed, radiological lung lesions and reduced long diffusion capacity are found in a large proportion of COVID-19 survivors, three months after contagion (Zhao et al. 2020; van den Borst 2021). However, long-COVID has been noted in patients with normal lung radiological aspect and function (Arnold et al. 2021). In addition to lungs, similar findings have been evidenced for brain radiological aspect and metabolism three months after acute COVID-19 (Lu et al. 2020). Another hypothesis is sustained pathological inflammation. Some patients remain positive for SARS-CoV-2 by polymerase chain reaction (PCR) for extended durations, up to four months (Li et al. 2020; Hirotsu et al. 2021).

Persistent viral replication could hypothetically trigger an immune activation which could be associated with long-COVID symptoms. Numerous studies have shown signs of immune alteration in COVID-19 or long-COVID: T-cell dysfunction, B-cell dysfunction, lymphopenia, and elevated pro-inflammatory markers, such as interleukin-6, ferritin, or D-dimer (Fathi and Rezaei 2020; Karlsson et al. 2020; Zuo et al. 2020; D’Amato et al. 2021). One last pathophysiological explanation is gut microbiome disruption, or gut dysbiosis (Yeoh et al. 2021).

Sleep disturbances and insomnia have been reported in both acute phase COVID and long-COVID. In a web-based study in China, 18.1% of participants reported poor sleep during the outbreak (Huang and Zhao 2020). This was reported as well by health-care professionals (Zhang et al. 2021). Another study in China found a 70% prevalence of insomnia symptoms at least once a week (Xue et al. 2020). Other reports have shown increased sleep disturbances during the COVID-19 outbreak (Xiao et al. 2020; Targa et al. 2021). Our group carefully surveyed the general population of France at the beginning of the lockdown as part of the COCONEL survey (Léger et al. 2020; Beck et al. 2021a, 2021b). It was observed at the beginning of the pandemic that a high prevalence of sleep disorders in the first sample of 1005 subjects (Beck et al. 2021a). For 62% of the people reporting sleep disorders in the survey, poor sleep was associated with some daytime impairment of daily activities. Such impairment mostly affected young people, most disadvantaged households, and the unemployed.

Sleep impairments, fatigue, and insomnia have been described in long-COVID but studies so far have focussed on qualitative and subjective analyses. A meta-analysis revealed a 31% prevalence of insomnia among long-COVID patients (Premraj et al. 2022). A case-series from Germany evidenced an association between insomnia and long-COVID in four patients (Young 2022). Recently Merikanto et al. confirmed that insomnia, fatigue, and excessive daytime sleepiness were among the most commonly reported symptoms in long-COVID, especially in patients with a history of severe acute COVID-19 (Merikanto et al. 2022).

Most studies so far on sleep and long-COVID were based on subjective parameters, with the exception of one study by Mekhael et al. using wearables (Mekhael et al. 2022). They showed, in patients with a history of COVID, decreased ‘light sleep’ and ‘deep sleep’, based on parameters, such as heart rate, heart rate variability, respiratory rate, and oxygen saturation.

The present study is the first to our knowledge to evaluate long-COVID patients by polysomnography, which is the current gold standard for sleep analysis. Polysomnography allows one to study sleep architecture, including the evaluation of the different sleep stages, as well as to evaluate the presence of sleep-related breathing disorders or abnormal movements (periodic limb movement disorder).

First, we analysed polysomnographic parameters of long-COVID patients and compared them to normal values (Boulos et al. 2019). The altered results are consistent with chronic insomnia in terms of short sleep duration, poor sleep efficiency, and high WAS indicating sleep maintenance insomnia. We, therefore, confirm the existence of objective insomnia in all the patients who complain of insomnia. It has been suggested that neurocognitive disorders associated with long-COVID could be related to a ‘brain fog’ phenomenon, which is seen in other conditions (Kverno 2021). In our group, 76% of patients complained of poor concentration and 53% of memory loss. Brain fog has been associated with abnormal REM sleep (REM-sleep without atonia) (Gagliano et al. 2021). Similar phenomena were not evidenced in our cohort, with normal REM sleep duration, proportion, and normal associated atonia.

Secondly, we compared polysomnographic results of long-COVID patients to those of matched insomniac controls. Those controls were initially consulted for chronic insomnia and may or may not have a history of acute COVID, but did not present with signs and symptoms consistent with long-COVID. We show that insomnia related to long-COVID is not different, on polysomnographic grounds, than chronic insomnia of controls (except for apnoea hypopnoea indexes, with however similar oxygen desaturation indexes) and with a lower percentage of N3. This could have several implications: (1) On pathophysiological level, those results suggest a common mechanism for ‘common’ chronic insomnia and long-COVID insomnia; (2) on a therapeutic level, those findings suggest that insomnia related to long-COVID should be treated like ‘common’ insomnia. The first line treatment for chronic insomnia is cognitive behavioural therapy (Riemann et al. 2017). In the case of insomnia symptoms associated with other medical conditions (such as depression or other chronic diseases), it is recommended to treat both, as the causal association and direction of the relationships are difficult to detangle.

Additionally, we performed more in-depth data regarding PSG parameters and clinical signs or symptoms, concerning both acute COVID and long-COVID. Owing to the relatively low number of subjects, most of the results were not statistically significant (data not shown). We did however show a positive association between the presence of arthralgias/enthesopathies in long-COVID and a shorter total sleep time (301 min ± 66.5 *vs.* 389 ± 62.5, *p* = 0.016). This is likely to be related to pain-induced impaired sleep. Besides, we show a positive association between memory loss in long-COVID and a decreased proportion of REM sleep (13.9% ± 5.7 *vs.* 23.8% ± 8.6, *p* = 0.017). This might be explained by the role of REM in memory acquisition and processing.

Besides physio pathological hypotheses, several simplest causes may have promoted insomnia associated with the changes in sleep habits and sleep hygiene linked to chronic diseases, like long-covid: lack of exercise, extended time in bed, increased time facing medias (Riemann et al. 2017).

We recognise several limitations to our findings: first, the small group of subjects limits the possibility of discussion. Second, we did not perform at this step micro or spectral analyses of PSG.

However, we emphasise that behind the complaints of poor sleep claimed by the patients with long-covid, it may have authentic and objective insomnia patients that have to be followed by sleep specialists.

## Supplementary Material

Supplemental Material
