# Supplementary material for: Polysomnographic parameters in long-COVID chronic insomnia patients
Source: Dialogues Clin Neurosci. 2023 Jun 30;25(1):43–9. doi: 10.1080/19585969.2023.2222714 (PMC10316734; doi:10.1080/19585969.2023.2222714)
Supplement: Supplemental Material [file TDCN_A_2222714_SM3705.docx]

**Appendices:**

**Supplementary table S1. Frequency of signs and symptoms presented by long-Covid patients in acute and chronic phase (n=17)**

|  |  | yes | no |
| --- | --- | --- | --- |
| **Signs and symptoms during inaugural Covid episode (acute phase)** | Neurological signs | 9 | 8 |
|  | Cardiothoracic signs | 16 | 1 |
|  | Digestive signs | 11 | 6 |
|  | ORL signs | 11 | 6 |
|  | Anosmia | 8 | 9 |
|  | Ageusia | 4 | 13 |
|  | Arthralgias | 2 | 15 |
|  | Myalgias | 5 | 12 |
|  | Dyspnea | 9 | 8 |
|  | Tachycardia | 3 | 14 |
|  | Headache | 6 | 11 |
| **Treatment received** | Hospitalization | 1 | 16 |
|  | Reanimation | 1 | 16 |
|  | Oxygen | 2 | 15 |
| **Symptom-free interval** | | 10 | 7 |
| **Signs and symptoms during long-Covid (chronic phase)** | Asthenia | 15 | 2 |
|  | Fever | 1 | 16 |
|  | Myalgias | 5 | 12 |
|  | Arthralgias Enthesopathies | 7 | 10 |
|  | Neurological signs | 17 | 0 |
|  | Headache | 7 | 10 |
|  | Vertigo | 4 | 13 |
|  | Neurogenic pain | 3 | 14 |
|  | Contractures, spasms | 3 | 14 |
|  | Difficulty focusing | 13 | 4 |
|  | Memory loss | 9 | 8 |
|  | Mood disorder | 6 | 11 |
|  | Cardiothoracic  signs | 16 | 1 |
|  | Dyspnea | 11 | 6 |
|  | Thoracic pain | 6 | 11 |
|  | Cough | 3 | 14 |
|  | Orthostatic hypotension | 3 | 14 |
|  | Cutaneomucosal signs | 8 | 9 |
|  | Digestive signs | 10 | 7 |
|  | ORL signs | 12 | 5 |
|  | Smell disorders | 8 | 9 |
|  | Ageusia | 2 | 15 |
|  | Ophthalmological disorders | 7 | 10 |

**Supplementary table S2. Biological markers of long Covid patients during the acute phase of the disease**

|  | Leukocytes  /mm^3^ | Lymphocytes  /mm^3^ | CRP  mg/L | ALT  IU/L | AST  IU/L | Ferritin  ng/mL | D-dimer  µg/L | TSH  mIU/L | ANA  IU/ml |
| --- | --- | --- | --- | --- | --- | --- | --- | --- | --- |
| Mean  (min – max) | 6,401  (3,700 – 10,950) | 2,197  (1,200 – 4,095) | 3.1  (0.3 – 20.4) | 27.9  (10 – 89) | 35.5  (12 – 196) | 124  (14 – 490) | 837  (0.57 – 2402) | 2.52  (0.48 – 8.86) | 145  (10 – 400) |
| Normal values | 4,000 – 11,000 | 1,000 – 4,800 | <5 | <49 | <34 | 10 - 291 | <500 | 0.55 – 4.78 | <7 |
| Missing values | 1 | 1 | 3 | 3 | 3 | 4 | 8 | 4 | 7 |

CRP: C-reactive protein; ALT: alanine aminotransferase; AST: aspartate aminotransferase; TSH: thyroid stimulating hormone; ANA: antinuclear antibodies
